# Qingre Yiqi Method along with Oral Hypoglycemic Drugs in Treating Adults with Type 2 Diabetes Mellitus: A Systematic Review and Meta-Analysis

**DOI:** 10.1155/2021/4395228

**Published:** 2021-09-11

**Authors:** Li Jiang, Shidong Wang, Jinxi Zhao, Weijun Huang, Jiayue Li, Yonghua Xiao, Hua Zhang, Qiang Fu, Yu Chen, Tao Yang, Esther Aijia Shen, Guanxun Su, Yaofu Zhang, Zhuang Li

**Affiliations:** ^1^Graduate School, Dongzhimen Hospital Affiliated to Beijing University of Chinese Medicine, Beijing, China; ^2^Section II of Endocrinology & Nephropathy Department of Dongzhimen Hospital Affiliated to Beijing University of Chinese Medicine, Beijing, China; ^3^Key Laboratory of Chinese Internal Medicine of Ministry of Education and Beijing, Dongzhimen Hospital Affiliated to Beijing University of Chinese Medicine, Beijing, China

## Abstract

**Objective:**

To evaluate the efficacy of the Qingre Yiqi method in the treatment of type 2 diabetes mellitus (T2DM) with meta-analysis.

**Method:**

The randomized controlled trials (RCTs) of the Qingre Yiqi method in the treatment of T2DM in the PubMed, Medline, EMBase, Cochrane Library, Web of Science, Weipu Journal, China Knowledge Network (CNKI), and Wanfang database were conducted. Three reviewers independently conducted the screening, extracted the data, and assessed methodological quality. Data analysis was performed using Rev Man 5.3 software for statistical analysis.

**Results:**

A total of 15 RCTs, including 1440 patients, were included. The results showed that compared with oral hypoglycemic drugs alone, the add-on treatment of the Qingre Yiqi method could significantly improve Chinese medicine syndrome (OR (95%CI) = 3.66 [2.47,5.42], *P* < 0.00001) and lower the level of HbA1c (MD (95%CI) = −0.68 [0.91, −0.45], *P* < 0.00001), triglyceride (TG) (MD (95%CI) = −0.38 [−0.58,-0.17], *P*=0.0004), low-density lipoprotein cholesterol (LDL-C) (MD (95%CI) = −0.25 [−0.37, −0.13], *P* < 0.0001), and total cholesterol(TC) (MD(95%CI) = −0.40[−0.67, −0.13], *P*=0.003). In terms of fasting blood glucose (FBG) and postprandial blood sugar (PBG), subgroup analysis showed that the baseline of FBG and the number of combined oral hypoglycemic drugs of PBG were the major sources of heterogeneity.

**Conclusion:**

Compared with the standard treatment, the Qingre Yiqi method along with oral hypoglycemic drugs showed the more beneficial effects for T2DM on improving TCM syndromes and reducing the blood glucose and partial lipid parameter.

## 1. Introduction

T2DM is an important public health problem in China. According to the 2017 survey by the China's National Center for Disease Control and Endocrinology Branch of the Chinese Medical Association, the prevalence rate of T2DM in China is 12.8% [1]. The complications of diabetes involve systemic tissues and organs, especially the damage to the eyes, kidneys, cardiovascular system, and nervous system, which seriously threatens the life quality of patients and brings heavy burden to the society [2].

As a special kind of traditional medicine established on over two thousand years of clinical practice, traditional Chinese medicine (TCM) has built a complete theoretical system of diagnosis and treatment. Ancient Chinese scholars noted that the physiological substances of the human body consisted of Qi and blood. The excessive endocrine and metabolic activities of the human body were regarded as internal heat. TCM considers pathogenesis of DM as “internal heat damages *Qi*.” Therefore, the Qingre Yiqi method, which means clearing the internal heat and supplementing *Qi*, is often applied in the treatment of DM in Chinese medicine [3]. Herb is an important part of TCM. Usually, herbs that are characterized with supplementing Qi include *Astragalus*, yam, *Poria*, *Atractylodes*, and ginseng, whose extracts have been proved by experiments to effectively reduce blood glucose and blood lipid [4], protect the function of the pancreatic islet [5], enhance glucose tolerance, and reduce glucagon secretion in rats [6]. Herbs that are characterized with clearing heat include *Coptis*, rhubarb, Radix Scutellariae, *Sophora flavescens*, and honeysuckle, whose extracts have also been proved to inhibit endoplasmic reticulum oxidative stress [7], alleviate intestinal inflammatory response [8], reduce the release of peripheral inflammatory cytokines [9], and alleviate insulin resistance [10]. A number of clinical studies have applied the Qingre Yiqi method on the basis of oral hypoglycemic drugs to relieve clinical symptoms and strengthen the hypoglycemic effect in T2DM. However, owing to variation in the sample size and methodological quality of the studies, the efficacy of the Qingre Yiqi method in adjuvant treatment for T2DM was still not fully understood. Therefore, the systematic review and meta-analysis were conducted to provide evidence.

## 2. Materials and Methods

### 2.1. Search Strategy

We followed the methods of Chen et al. [11]. Eight databases (PubMed, EMBase, Cochrane Library, Web of Science, Weipu Journal, China Knowledge Network (CNKI), and Wanfang database) were searched for patients of T2DM. Clinical studies published before February 2021 were retrieved by combining subject words with free words and linking the corresponding Boolean logical operators. The following domains of terms were used: “Type 2 diabetes,” “TCM,” “clear Heat,” “supplement Qi, ”“Replacement therapy,” and “Qingre Yiqi.” See Supplemental File 1 for a full description of the search strategy (Supplemental File 1 search strategy). There was no restriction on language or study design. The Google and Baidu academic database were also searched for potential relevant articles. A protocol for the systematic review and meta-analysis has been registered in the PROSPERO (CRD42021253901).

### 2.2. Including and Excluding Criteria

#### 2.2.1. Including Criteria

We included studies that met the following inclusion criteria: (1) types of studies: randomized controlled trials (RCTs); (2) type of participants: patients diagnosed with T2DM either using 2020 CDS Guidelines for the Prevention and Treatment of T2DM [1] or 2020 American Diabetes Association diagnostic criteria [12]; (3) main intervention: the control group was treated with oral hypoglycemic drugs, while the experimental group was additionally treated with a TCM prescription based on the Qingre Yiqi method. The definition of the Qingre Yiqi method: ① the names of the formula include “Qingre Yiqi” or “supplement *Qi* and clear Heat” or ② the total proportion of the two kind of drugs should be greater than or equal to 50%. The representatives of heat-clearing drugs are *Coptis*, rhubarb, Radix Scutellariae, *Sophora flavescens*, and honeysuckle, while the representatives of Qi-supplementing drugs are *Astragalus*, yam, *Poria*, *Atractylodes*, and ginseng; and (4) outcomes which included HbA1c, FBG, PBG, fasting insulin(FIL), TG, TC, high-density lipoprotein cholesterol (HDL-C), LDL-C, C-reactive protein (CRP), and TCM syndromes.

#### 2.2.2. Excluding Criteria

We excluded trials that met the following exclusion criteria: (1) studies that did not have required outcomes; (2) studies with incomplete general data and baseline indicators; (3) studies that did not have standardized control or the control group did not follow guidelines; (4) formulas that were not consistent with the definition of the Qingre Yiqi method; (5) the target population which was inconsistent with diagnostic criteria of T2DM; and (6) the study with duplicate publication.

### 2.3. Data Abstraction and Quality Assessment

The quality assessment team consisted of three members. Two members conducted the initial screening of the literature and marked the fuzzy literature according to the inclusion and exclusion criteria. The other member reviewed and determined the final inclusion literature. The extracted data included the first author(s), location, size and age of sample, course of disease, interventions details, outcomes, follow-up periods, and adverse events. The Cochrane manual correction formula was used to correct the data in the original literature [13]:

(1) $$\begin{array}{c} \text{Mean}\left(C\right)=\text{mean}\left(F\right)-\text{mean}\left(B\right),\\ {\text{SD}}_{1}\left(C\right)=\sqrt{{\text{SD}}_{1}{\left(B\right)}^{2}+{\text{SD}}_{1}{\left(F\right)}^{2}-\left(2\times R_{1}\times {\text{SD}}_{1}\left(B\right)\times {\text{SD}}_{1}\left(F\right)\right)}. \end{array}$$

*B* refers to baseline, *F* refers to final, and *R*_1_ = 0.5. The guiding principles for clinical research of new Chinese medicine in 2002 were referred to examine the efficacy criteria [14]. The standard ROB bias risk assessment tool was used in the assessment of bias risk [15], with appropriate reference to the assessment method published in the Cochrane Library [13], which contains evaluation of randomization, allocation concealment, blinding of participants and outcome, incomplete outcome data, selective reporting, withdrawals and dropouts, and other biases.

### 2.4. Statistical Analysis

ReviewManager5.3 software was used for statistical analysis. The continuous variable outcomes and dichotomous outcomes were analyzed using mean difference (MD) and the risk ratio (RR), both of which were given 95% confidence intervals. The Cochrane *Q* test was used to analyze the heterogeneity among studies. *P* < 0.10 indicated that the heterogeneity among studies was statistically significant. In addition, *I*^2^ statistic was used to quantitatively evaluate the magnitude of heterogeneity according to the chi-square test. Under the premise of good condition of clinical homogeneity, when the *I*^2^ was <25%, the fixed-effect model was used. When the *I*^2^ lied between 25% ∼ 75%, the random-effect model was chosen. When the *I*^2^ was>75%, the sources of heterogeneity would be determined by sensitivity analysis, subgroup analysis, and meta-regression. If the heterogeneity was still high, only descriptive statistics were conducted [16].

## 3. Results

### 3.1. Search Results

**Figure 1:**
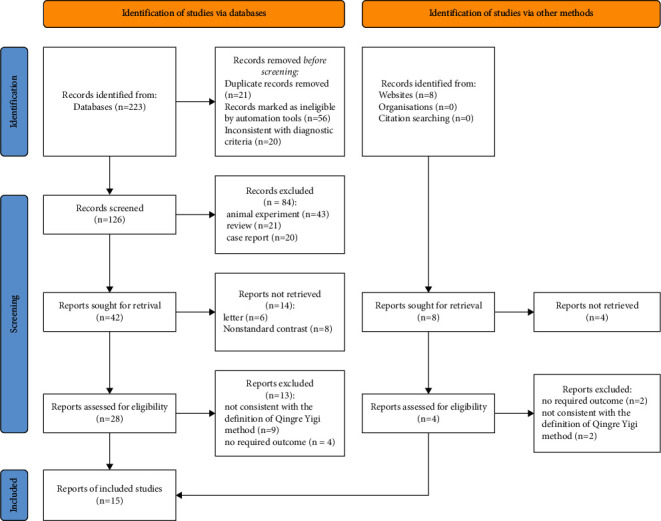
Flow diagram of the included studies for this meta-analysis.

A total of 231 potential articles up to February 2021 (223 from databases and 8 from websites) were identified with the electronic-based search. After removing duplicates and nonstandard diagnosis, 126 articles remained. We excluded 94 articles by screening titles and abstracts and retrieved the full texts of 32 remaining articles. Finally, 15 studies [17–31] met the inclusion criteria and were included in this meta-analysis (Figure 1).

### 3.2. Characteristics of the Eligible Studies

All studies included were RCTs, and the characteristics of these studies are summarized in Table 1. Studies were published from 2003 to 2019 and originated from China. The sample size of the 10 studies ranged from 40 to 243, and the course of treatment lasted from 4 to 24 weeks. The distribution of age and gender had no significant difference. Three studies reported follow-up [18, 23, 28], and two reported specific adverse events [20, 27].

### 3.3. Methodological Quality of Included Studies

**Figure 2:**
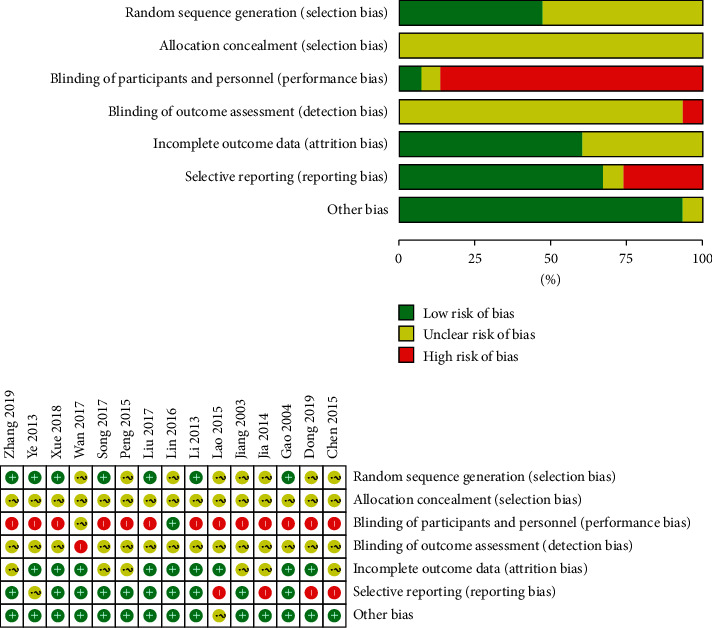
Risk of bias graph.

Only 7 studies have described the generation of random sequences [19, 20, 22–24, 26, 27], all of which were generated by random number tables, so the risk of bias was low. The remaining 8 studies only described the word “random” and did not describe or explain the specific method of random implementation, so it was difficult to judge the risk of bias. None of the studies mentioned the method of random concealment, so it was difficult to judge the allocation concealment. Only one of the studies mentioned blinding of the participants [18], but the implementation method was not reported. None of the studies mentioned the blinding of outcome assessment, but the team comprehensively evaluated the clinical study process and believed that the outcome might be less affected by the lack of the method. No data were missing in the included papers. Four studies were considered as selective reporting [16, 27–29], and no other bias existed (Figure 2).

### 3.4. Treatment Effects

#### 3.4.1. Primary Outcome

**Figure 3:**
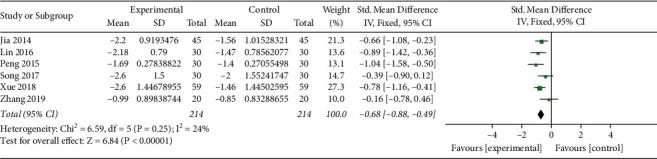
Forest plot of HbA1c.

*(i). HbA1c*. HbA1c levels were reported in 8 studies (*n* = 592) [19, 20, 22, 24–26, 28, 30]. In one of the trials [19], the basic oral hypoglycemic drug was thiazolidinedione, while in the remaining trials, it was metformin, so it was not combined for analysis. Another trial [28] did not make TCM syndrome diagnosis, while the rest of the reports have made TCM syndrome diagnosis according to guiding principles for clinical research of new Chinese medicine, so the combined analysis was also not performed. A total of 214 patients in the experimental group and 214 patients in the control group were included in the remaining 6 trials, with low heterogeneity among studies (*I*^2^ = 24%, *P*=0.25). Analysis of the fixed-effect model showed that MD (95%CI) = −0.68 [0.91, −0.45], *P* < 0.00001, which suggested that the Qingre Yiqi method along with oral hypoglycemic drugs took the favorable effects for decreasing HbA1c levels of T2DM (Figure 3).

#### 3.4.2. Secondary Outcomes

**Figure 4:**
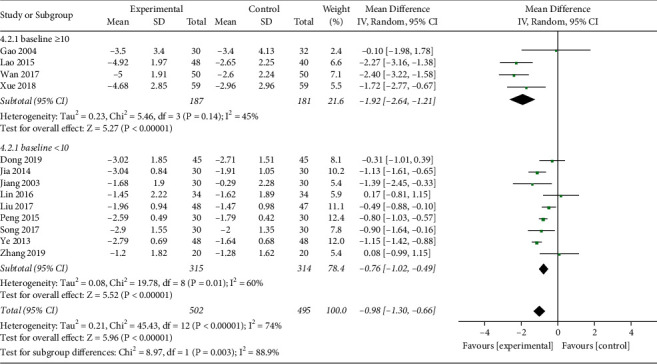
Forest plot of FBG.

*(i). FBG*. FBG levels were reported in 14 studies (*n* = 1197) [17–28, 30, 31]. One trial [24] was excluded for its high risk of bias in ROB assessment. A total of 502 patients in the experimental group and 495 patients in the control group were included in the remaining 13 trials, with moderate heterogeneity among studies (*I*^2^ = 74%, *P* < 0.00001). Sensitivity analysis showed that there was strong stability between the reports, and no major source of heterogeneity was found. Subgroup analysis was performed for the mean baseline of FBG ≥10 mmol/L and <10 mmol/. In 4 studies [17, 26, 27, 31], FBG baseline levels were, respectively, 10.16 ± 1.78, 11.87 ± 3.29, 10.9 ± 3.9, and 10.20 ± 1.79 mmol/*L*, which in the remaining 9 studies were all lower than 10 mmol/L. The heterogeneity analysis suggested that there was lower heterogeneity in both subgroups (*I*^2^ = 45%, *I*^2^ = 60%). Analysis of the random-effect model showed that MD = −1.92, 95%CI [−2.64, −1.21], *P* < 0.00001, in the subgroup of baseline ≥10 mmol/L, while in the subgroup of baseline <10 mmol/L, analysis of the random-effect model showed that MD = −0.76, 95%CI [−1.02, −0.49], *P* < 0.00001. The aggregated results suggested that the Qingre Yiqi method along with hypoglycemic drugs showed favorable effects for changing FBG levels of T2DM, while the magnitude of the decrease was related to FBG baseline (Figure 4).

**Figure 5:**
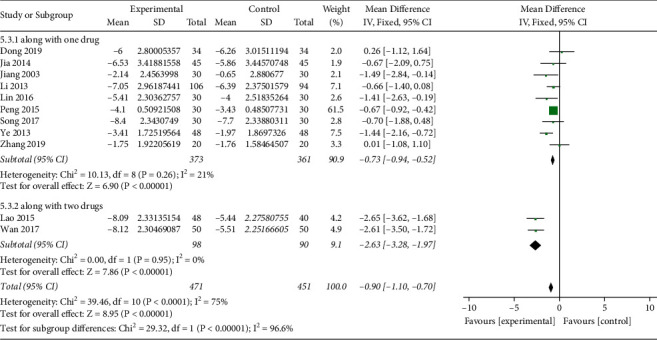
Forest plot of PBG.

*(ii). PBG*. PBG levels were reported in 11 studies (*n* = 922) [17–22, 24, 25, 28, 30, 31], including 471 patients in the treatment group and 451 patients in the control group. The heterogeneity test showed a high heterogeneity among the studies (*I*^2^ = 75%, *P* < 0.0001). Sensitivity analysis showed that there was strong stability between the reports, and the major source of heterogeneity was not found. Subgroup analysis was performed for the combined use of hypoglycemic drugs. In two studies [17, 31], the intervention measures in the experimental group were TCM plus metformin and gliclazide, which meant the combined use of two kinds of hypoglycemic drugs. In the remaining 9 studies, the combined use of hypoglycemic drugs only involved one kind of hypoglycemic drug regardless of metformin or gliclazide or pioglitazone or glipizide. The heterogeneity analysis suggested that there was much lower heterogeneity in both subgroups (*I*^2^ = 21%, *I*^2^ = 0%). Analysis of the fixed-effect model showed that MD = −0.73, 95%CI [−0.94, −0.52], *P* < 0.00001, in the subgroup of use of one hypoglycemic drug, while in the use of two kinds of hypoglycemic drug, analysis of the fixed-effect model showed that MD = −2.63, 95%CI [−3.28, −1.97], *P* < 0.00001. The aggregated results suggested that the Qingre Yiqi method along with hypoglycemic drugs showed favorable effects for changing PBG levels of T2DM, while the magnitude of the decrease was related to the number of combination drugs (Figure 5).

**Figure 6:**
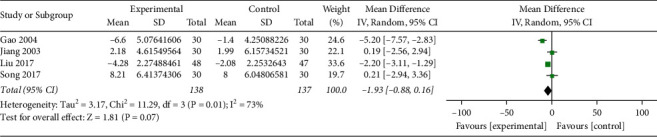
Forest plot of FIL.

*(iii). FIL*. FIL was reported in 4 studies (*n* = 275) [19, 21, 23, 27], including 138 patients in the treatment group and 137 patients in the control group. The heterogeneity test showed a moderate heterogeneity among the studies (*I*^2^ = 73%, *P*=0.01). Analysis of the random-effect model showed that MD = −1.93, 95%CI (−4.02, −0.16), *P*=0.07. The difference was not statistically significant, suggesting that the Qingre Yiqi method combined with hypoglycemic drugs did not show favorable effects than hypoglycemic drugs alone in reducing the fasting insulin secretion of T2DM (Figure 6).

**Figure 7:**
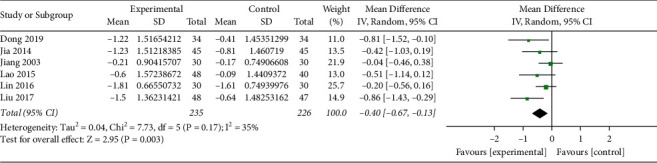
Forest plot of TC.

**Figure 8:**
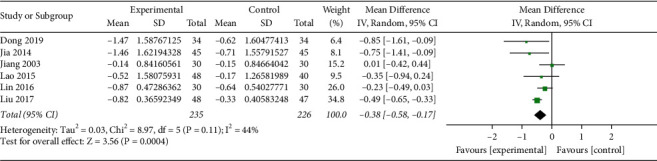
Forest plot of TG.

*(iv). Blood Lipid Levels*. TC and TG were reported in 7 studies (*n* = 792) [17, 18, 21, 23, 24, 28, 30], among which there was one trial stipulating that patients with dyslipidemia should not be treated with lipid-lowering drugs [24], while the others did not limit the use of lipid-lowering drugs. So, the trial was not combined for analysis. A total of 235 patients in the experimental group and 226 patients in the control group were included in the remaining 6 trials, with moderate heterogeneity among studies of TC (*I*^2^ = 35%, *P*=0.17) and moderate heterogeneity among studies of TG (*I*^2^ = 44%, *P*=0.11). Analysis of the random-effect model in TC showed that MD (95%CI) = −0.40 [−0.67, −0.13], *P*=0.003, and that in TG showed MD (95%CI) = −0.38 [−0.58, −0.17], *P*=0.0004, indicating that the Qingre Yiqi method along with oral hypoglycemic drugs showed the favorable effects for reducing TC and TG levels of T2DM (Figures 7 and 8).

**Figure 9:**
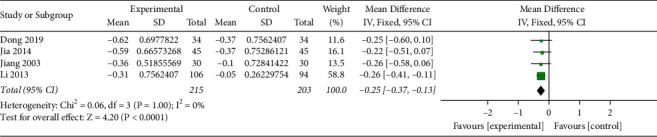
Forest plot of LDL-C.

LDL-C was reported in 5 studies (*n* = 513) [21, 23, 24, 28, 30], including 263 patients in the experimental group and 250 patients in the control group. The heterogeneity test showed a high heterogeneity among the studies (*I*^2^ = 78%, *P*=0.001). So, the sensitivity analysis was used to eliminate individual studies one by one. It was found that “Liu 2017” had a great influence on the combined effect size. In this trial, the drug dose of TCM in the experimental group was three times a day, while in other trials, the drug dose was once or twice a day. Therefore, the drug dose was considered as the source of heterogeneity. The heterogeneity test after elimination showed a low heterogeneity among the studies (*I*^2^ = 0%, *P*=1.00). Analysis of the fixed-effect model of LDL-C in the remaining 4 studies showed that MD (95%CI) = −0.25 [−0.37, −0.13], *P* < 0.0001, suggesting that the Qingre Yiqi method along with oral hypoglycemic drugs showed the favorable effects for reducing LDL-C levels of T2DM (Figure 9). HDL-C was reported in 3 studies [21, 23, 24], including 184 patients in the experimental group and 171 patients in the control group. The heterogeneity test showed a high heterogeneity among the studies (*I*^2^ = 77%, *P*=0.17). See Supplemental File 2 for a detailed figure of HDL-C (Supplemental File 2 forest plot of HDL-C). The source of heterogeneity was not found, so only descriptive analysis was conducted for this indicator. Among the 3 included studies, there were statistically significant differences in 2 studies [23, 24] compared with the experimental group before intervention (*P* < 0.05). There were statistically significant differences in 1 study [20] compared with the control group after intervention.

*(v). CRP*. CRP was reported in 4 studies (*n* = 418) [18, 20, 24, 27], including 214 patients in the experimental group and 204 patients in the control group. The heterogeneity test showed a high heterogeneity among the studies (*I*^2^ = 97%, *P* < 0.0001). See Supplemental File 3 for a detailed figure of CRP (Supplemental File 3 forest plot of CRP). Only descriptive analysis was conducted for this indicator. Among the 4 included studies, there were statistically significant differences in 3 studies [18, 20, 27] compared with the experimental group before intervention (*P* < 0.05). There were statistically significant differences in 1 study [24] compared with the control group after intervention.

**Figure 10:**
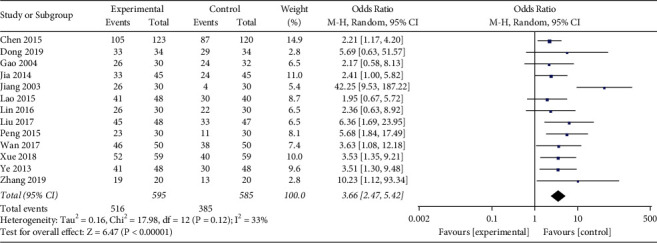
Forest plot of TCM syndromes.

*(vi). TCM Syndromes*. TCM syndromes were reported in 13 studies (*n* = 1128) [17, 18, 20–23, 25–31], including 595 patients in the experimental group and 585 patients in the control group. The heterogeneity test showed a moderate heterogeneity among the studies (*I*^2^ = 33%, *P*=0.12). Analysis of the random-effect model showed that OR (95%CI) = 3.66 [2.47,5.42], *P* < 0.00001, which suggested that the Qingre Yiqi method along with oral hypoglycemic drugs took the favorable effects for improving TCM syndromes of T2DM (Figure 10).

### 3.5. Adverse Events

Adverse events were observed in 5 studies [18, 20, 21, 27, 30], among which 3 reported that no adverse reactions appeared during the trial [21, 27, 30], 1 report that 6 patients showed high fever in the control group and 4 patients showed vomiting in the experimental group [18], and 1 reported that 1 case of hypoglycemia occurred in each of the two groups [20]. The other studies did not report adverse events.

### 3.6. Publication Bias

**Figure 11:**
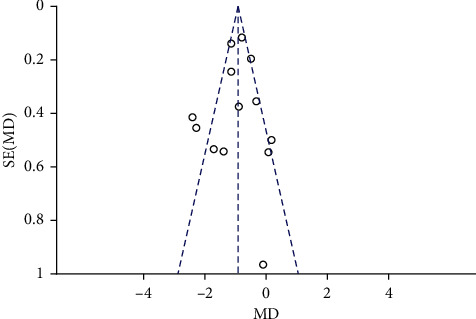
Publication bias of the funnel plot of FPG.

Meta-analysis was conducted after removing single studies for each effect index. The results showed that the effect value did not change significantly, suggesting that the results were relatively steady. The funnel plot of FBG is basically symmetric, indicating that there was no publication bias in these studies (Figure 11).

## 4. Discussion

This meta-analysis included 15 randomized clinical trials which applied the Qingre Yiqi method to the treatment of T2DM. All the trials had clear diagnostic criteria, inclusion criteria, and exclusion criteria. The results showed that compared with standard treatment of hypoglycemic drugs alone, the Qingre Yiqi method along with oral hypoglycemic drugs seemed to be more effective in treatment of T2DM by improving HbA1c, FBG, PBG, TG, TC, LDL-C, and TCM syndromes, but not for FIL. Because of the high heterogeneity, HDL-C and CRP were not to be analyzed.

According to the combined results, the Qingre Yiqi method along with oral hypoglycemic drugs took the favorable effects for decreasing HbA1c levels of T2DM (MD (95%CI) = −0.68 [0.91, −0.45]). The clinical threshold for T2DM was 6.5% [1]. In the included studies, except for the work of Zhang et al. [22], the baseline of treatment was all higher than 7%. After intervention, HbA1c levels in the control group did not return to the normal level, while it decreased below the threshold value under adjuvant therapy of the Qingre Yiqi method in five studies [18–20, 25, 26], suggesting the possible clinical significance of the Qingre Yiqi method. In terms of clinical symptoms, such as fatigue and thirst in the reported work, the Qingre Yiqi method could also provide a definite effect.

In addition, when the FBG baseline was ≥10 mmol/*L*, the Qingre Yiqi method could exhibit a better effect with another average drop of 1.92 mmol/L in FBG on the basis of standard treatment. When the baseline was <10 mmol/*L*, the Qingre Yiqi method could lower another 0.76 mmol/L in FBG on average. In terms of PBG, when combined with two kinds of hypoglycemic drugs, the Qingre Yiqi method could exhibit a better effect with another average drop of 2.63 mmol/L in PBG on the basis of standard treatment. When combined with one kind of hypoglycemic drug, it could lower another 0.73 mmol/L in PBG on average.

Previous studies showed that the Qingre Yiqi method mainly played an important role in the treatment of T2DM by reducing inflammatory cytokines [32], achieving resistance to oxidative stress and free radicals [33], increasing insulin sensitization, protecting the function of the islet [34], and regulating the structure of intestinal flora [35]. When fasting plasma glucose is higher than 10 mmol/L, the glucose toxicity is obvious, meaning the hyperactivity of tissue aerobic oxidation and glycolysis, leading to the result that the synthesis of *β* cells itself is not enough to complement the release of insulin, so the insulin in the cell pool is empty and insulin resistance appears [36]. The Qingre Yiqi method includes some herbs such as *Coptis*, rhubarb, Radix Scutellariae, and *Sophora flavescens*, which can effectively reduce the effects of oxidative stress and release of inflammatory factors, therefore alleviating the glucose toxicity [7, 8], which may explain the favorable effect of the Qingre Yiqi method when blood glucose baseline is high. In addition, as type 2 diabetes involves multiple pathological processes, such as increasing secretion of glucagon, decreasing secretion of insulin, weakness of incretin effect, reduced glucose uptake, and neurotransmitter dysfunction [37], combined use of multiple oral hypoglycemic drugs performs significantly better than the single use of the drugs. Rosenstock et al. have explored the efficacy and tolerability of initial combination therapy with vildagliptin and pioglitazone compared with component monotherapy. It was proved that treatment with the vildagliptin/pioglitazone combination in patients with T2DM provided better glycaemic control than either monotherapy component yet had minimal risk of hypoglycaemia and a tolerability profile comparable with component monotherapy [38], which may explain the good effect of the Qingre Yiqi method in combination with two hypoglycemic drugs. The result of meta-analysis showed the Qingre Yiqi method did not have a significant effect on FIL, which was not consistent with the animal experiment [39]. It is probably because that the disease model in the animal experiment was prediabetes, not type 2 diabetes with long duration and obvious insulin resistance, which means more sensitivity to the Qingre Yiqi method.

However, this meta-analysis was limited by the following factors: (1) The meta-analyses only included 15 studies assessing a total of 1440 patients, which did not represent the general characteristics of the sample. (2) The included studies were not of very high quality and all were in China. (3) Most studies did not report an adverse event. A few reported the occurrence of adverse events, but the events were not classified and the corresponding treatment measures were not described. (4) Most studies did not conduct follow-up or the descriptions of follow-up were too brief. (5) Even if the connotation of the Qingre Yiqi method was strictly defined, heterogeneity was inevitable due to the different compositions of the formula. Based on the abovementioned limitations, it is difficult to draw a definite conclusion. Therefore, in future clinical studies, large-sample, high-quality, multicenter, multilevel, and properly-blinded randomized controlled trials are required to be carried out. Only by following the evidence-based medicine theory and conducting experiments under the unified standard can we improve the quality of meta-analysis and verify the effectiveness of the Qingre Yiqi method of TCM.

## 5. Conclusions

In conclusion, our systemic review initially demonstrated the therapeutic effects of the Qingre Yiqi method in T2DM patients. Due to the limitation of this meta-analysis, more high-quality RCTs are expected to be conducted to provide more accurate clinical evidence.

## Figures and Tables

**Table 1:** Characteristics of the eligible studies.

Studies (first author, year)	Location	Patient no. (T/C)	Gender, male/female	Mean age (*x* ± *s*, years)	Course of disease (*x* ± *s*, years)	Treatment group	Control group	Course of treatment (week)	Follow-up (week)	Outcome
Lao [17], 2015	China	48/40	T:30/22 C:24/22	T:53.16 ± 24.31 C:56.21 ± 21.81	T:7.64 ± 6.27 C:8.16 ± 6.42	TCM + metformin + gliclazide	Metformin + gliclazide	12	NMT	①⑤⑥⑦
Lin [18], 2016	China	30/30	T:13/17 C:16/14	T:74.70 ± 6.10 C:74.97 ± 5.70	NMT	TCM + metformin	Metformin	4	4	①②③⑤⑥⑦⑩
Song et al. [19], 2017	China	30/30	T:14/16 C:15/15	T:55.1 ± 12.4 C:56.0 ± 11.4	NMT	TCM + metformin	Metformin	12	NMT	①②③④
Ye et al. [20], 2013	China	48/48	T:28/20 C:26/22	T:54.9 ± 1.8 C:55.3 ± 2.4	T:7.9 ± 1.1 C:7.8 ± 1.0	TCM + pioglitazone	Pioglitazone	8	NMT	①②③⑤⑩
Jiang [21], 2003	China	30/30	T:18/12 C:17/13	NMT	NMT	TCM + glipizide	Glipizide	8	NMT	①②④⑤⑥⑦⑧⑨
Zhang et al. [22], 2019	China	20/20	T:11/9 C:8/12	T:52.55 ± 7.54 C:51.85 ± 7.84	NMT	TCM + metformin	Metformin	12	NMT	①②③⑤
Liu et al. [23], 2017	China	49/47	T:33/15 C:31/16	T:52.85 ± 6.32 C:54.37 ± 6.45	T:5.74 ± 2.04 C:5.31 ± 1.17	TCM + metformin	Metformin	8	8	①②④⑤⑥⑦⑧⑨
Li et al. [24], 2013	China	106/94	T:56/50 C:50/54	T:43.61 ± 8.21 C:44.57 ± 7.38	NMT	TCM + pioglitazone	Pioglitazone	4	NMT	①②⑥⑦⑧⑨⑩
Peng et al. [25], 2015	China	30/30	T:13/17 C:16/14	T:49.8 ± 7.48 C:50.5 ± 7.73	NMT	TCM + metformin	Metformin	12	NMT	①②③⑤
Xue [26], 2018	China	59/59	T:36/23 C:34/25	T:56.18 ± 6.72 C:55.72 ± 6.41	NMT	TCM + metformin	Metformin	8	NMT	①③⑤
Gao et al. [27], 2004	China	30/32	T:18/14 C:14/16	T:63.8 ± 9.2 C:65.6 ± 7.3	T:4.8 ± 5.1 C:5.2 ± 3.2	TCM + sulfonylureas	Sulfonylureas	4	NMT	①④⑤⑩
Dong [28], 2019	China	34/34	T:21/13 C:19/15	T:70.29 ± 4.24 C:69.59 ± 5.25	T:9.73 ± 5.34 C:7.69 ± 4.23	TCM + metformin	Metformin	24	12	①②③④⑤⑦⑧⑨
Chen and He [29], 2015	China	123/120	T:63/60 C:64/56	T : 61 C:58.3	NMT	TCM + metformin	Metformin	12	NMT	⑤
Jia and Zhao [30], 2014	China	45/45	NMT	60.5 ± 5.1	5.2 ± 1.7	TCM + metformin/sulfonylureas	Metformin/sulfonylureas	12	NMT	①②③⑤⑥⑦⑧⑨
Jia and Ahao [30], 2017	China	50/50	T:28/22 C:30/20	T:56.8 ± 8.0 C:55.0 ± 8.5	T:7.8 ± 5.0 C:8.0 ± 6.5	TCM + metformin + gliclazide	Metformin + gliclazide	4	NMT	①②⑤⑥⑦⑧

NMT: not mentioned. T: treatment group, C: control group. ① FPG, ② PBG, ③ HbA1c, ④ INS, ⑤ TCM syndromes, ⑥ TG, ⑦ TC, ⑧ HDL-C, ⑨ LDL-C, and ⑩ CRP.

## Data Availability

No data were used to support this study.

## Supplementary Materials

Supplementary Materials Supplementary Description: 1. Supplemental File 1 search strategy in PubMed. 2. Supplemental File 2 forest plot of HDL-C. 3. Supplemental File 3 forest plot of CRP.
