# Supplementary material for: Qingre Yiqi Method along with Oral Hypoglycemic Drugs in Treating Adults with Type 2 Diabetes Mellitus: A Systematic Review and Meta-Analysis
Source: Evid Based Complement Alternat Med. 2021 Sep 11;2021:4395228. doi: 10.1155/2021/4395228 (PMC8452389; doi:10.1155/2021/4395228)
Supplement: Supplementary Materials — Supplementary Description: 1. Supplemental File 1 search strategy in PubMed. 2. Supplemental File 2 forest plot of HDL-C. 3. Supplemental File 3 forest plot of CRP. [file 4395228.f1.zip › 4395228.f1/Supplemental File 1 description of search strategy.pdf]

Supplemental File 1 Search strategy in PubMed

search strategy	[PubMed]
Type 2 Diabetes Mellitus	#1 “Diabetes Mellitus, Type 2”[Mesh] OR “Diabetes Mellitus, Noninsulin-Dependent*”[All Fields] OR “Diabetes Mellitus, Ketosis-Resistant*”[All Fields] OR “Non-Insulin-Dependent Diabetes Mellitus*”[All Fields] OR “NIDDM*”[All Fields] OR “Diabetes Mellitus, Maturity-Onset*” OR “Maturity-Onset Diabetes Mellitus*”[All Fields] OR “Diabetes Mellitus, Slow-Onset”[Mesh] or “Type 2 Diabetes*, Slow Onset”[All Fields] or“Diabetes Mellitus, Adult Onset”[All Fields] or “Adult-Onset Diabetes Mellitus”[All Fields] or “Diabetes, Maturity-Onset*”[All Fields] or “Maturity Onset Diabetes*”[All Fields]
intervention	#2 “Drugs, Chinese Herbal” [Mesh] OR “Chinese Drugs, Plant” [All Fields] OR “Chinese Herbal Drugs” [All Fields] OR “Herbal Drugs, Chinese” [All Fields] OR “Plant Extracts, Chinese” [All Fields] OR “Chinese Plant Extracts” [All Fields] OR “Extracts, Chinese Plant” [All Fields]
	#3 “clear Heat”[All Fields] OR “clearing heat, Plant”[All Fields] OR “Heat-clearing Herbal Drugs” [All Fields] OR “clear away heat[All Fields] OR “alleviate heat”[All Fields] OR “clear fire”[All Fields] OR “Qing Re”[All Fields]
	#4 “supplement Qi”[All Fields] OR “tonifying Qi”[All Fields] OR “nourish Qi”[All Fields]OR “Yi Qi”[All Fields]
	#5 “Coptis”[Mesh] OR “Goldthread* ”[All Fields] OR “Goldthreads* ”[All Fields] OR “Panax ginseng”[Mesh] OR “Korean Ginsengs*”[All Fields] OR “Ginseng, Korean*”[All Fields] OR “Korean Ginseng”[All Fields] OR “Red Ginseng, Korean”[All Fields] OR “Schinsengs”[All Fields] OR “Jen Shen”[All Fields] OR “Shinsengs”[All Fields] OR “Shinseng”[All Fields] OR “Renshens”[All Fields] OR “Renshen”[All Fields] OR “Ninjins”[All Fields] OR “Ninjin”[All Fields]
intervention	#6 “Treatment*”[All Fields] OR Therapy[All Fields] OR Therapies[All Fields]
Study	#7 “randomized controlled trial”[pt] OR “controlled clinical trial”[pt] OR” randomized”[tiab] OR randomised[tiab] OR randomly[tiab] OR randomization[tiab] OR randomisation[tiab] OR random allocation[mh] OR placebo[mh] OR placebo[tiab] OR trial[tiab] OR groups[tiab]
Final Search	#8 #1AND (#2 OR #3 OR #4 OR #5 OR #6) AND #7

Clinical studies published before February 2021 were retrieved by combining subject words with free words and linking corresponding Boolean logical operators.
