# Supplementary material for: Qingre Yiqi Method along with Oral Hypoglycemic Drugs in Treating Adults with Type 2 Diabetes Mellitus: A Systematic Review and Meta-Analysis
Source: Evid Based Complement Alternat Med. 2021 Sep 11;2021:4395228. doi: 10.1155/2021/4395228 (PMC8452389; doi:10.1155/2021/4395228)
Supplement: Supplementary Materials — Supplementary Description: 1. Supplemental File 1 search strategy in PubMed. 2. Supplemental File 2 forest plot of HDL-C. 3. Supplemental File 3 forest plot of CRP. [file 4395228.f1.zip › 4395228.f1/Supplemental File 2 description of forest plot of HDL-C.pdf]

Supplemental File 2 Forest plot of HDL-C

	Experimental			Control				Mean Difference
Study or Subgroup	Mean	SD	Total	Mean	SD	Total	Weight	IV, Random, 95% CI
Jiang 2003	0.07	0.29638578	30	0.06	0.36879817	30	29.7%	0.01 [-0.16, 0.18]
Li 2013	0.13	0.44237993	106	-0.06	0.58025856	94	32.5%	0.19 [0.05, 0.33]
Liu 2017	0.5	0.24879711	48	0.2	0.23515952	47	37.8%	0.30 [0.20, 0.40]
**Total (95% CI)**			**184**			**171**	**100.0%**	**0.18 [0.02, 0.34]**

Heterogeneity: $Tau^2 = 0.02$; $Chi^2 = 8.67$, df = 2 (P = 0.01); $I^2 = 77\%$
Test for overall effect: Z = 2.15 (P = 0.03)

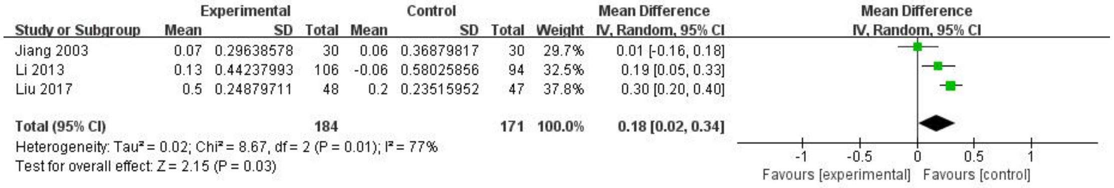

HDL-C was reported in 3 studies, including 184 patients in the experimental group and 171 patients in the control group. The heterogeneity test showed a high heterogeneity among the studies(I2=77%, P=0.17) .Therefore, only descriptive analysis was conducted.
