# Supplementary material for: Qingre Yiqi Method along with Oral Hypoglycemic Drugs in Treating Adults with Type 2 Diabetes Mellitus: A Systematic Review and Meta-Analysis
Source: Evid Based Complement Alternat Med. 2021 Sep 11;2021:4395228. doi: 10.1155/2021/4395228 (PMC8452389; doi:10.1155/2021/4395228)
Supplement: Supplementary Materials — Supplementary Description: 1. Supplemental File 1 search strategy in PubMed. 2. Supplemental File 2 forest plot of HDL-C. 3. Supplemental File 3 forest plot of CRP. [file 4395228.f1.zip › 4395228.f1/Supplemental File 3 description of forest plot of CRP.pdf]

Supplemental File 3 Forest plot of CRP

	Experimental			Control				Mean Difference
Study or Subgroup	Mean	SD	Total	Mean	SD	Total	Weight	IV, Random, 95% CI
Gao 2004	-4.4	1.35277493	30	-0.3	1.3114877	32	22.2%	-4.10 [-4.76, -3.44]
Li 2013	-2.04	0.23388031	106	-1.58	0.26888659	94	33.4%	-0.46 [-0.53, -0.39]
Lin 2016	-3.05	2.59497592	30	-0.99	2.59223842	30	11.2%	-2.06 [-3.37, -0.75]
Ye 2013	-2.06	0.24248711	48	-1.52	0.26153394	48	33.2%	-0.54 [-0.64, -0.44]
**Total (95% CI)**			**214**			**204**	**100.0%**	**-1.47 [-2.01, -0.94]**

Heterogeneity: Tau² = 0.22; Chi² = 119.91, df = 3 (P < 0.00001); I² = 97%
Test for overall effect: Z = 5.38 (P < 0.00001)

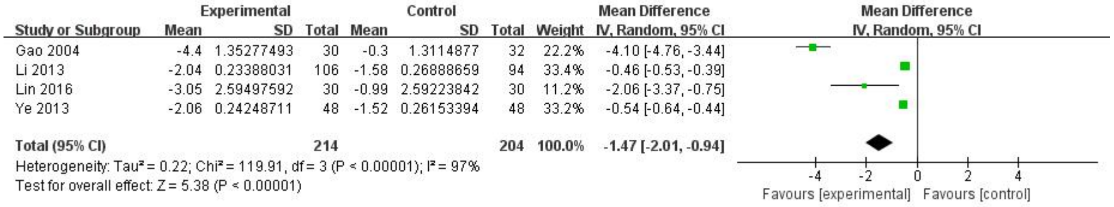

CRP was reported in 4 studies (n=418), including 214 patients in the experimental group and 204 patients in the control group. The heterogeneity test showed a high heterogeneity among the studies(I2=97%,P<0.0001). Therefore, Only descriptive analysis was conducted.
