# Supplementary material for: Qingre Yiqi Method along with Oral Hypoglycemic Drugs in Treating Adults with Type 2 Diabetes Mellitus: A Systematic Review and Meta-Analysis
Source: Evid Based Complement Alternat Med. 2021 Sep 11;2021:4395228. doi: 10.1155/2021/4395228 (PMC8452389; doi:10.1155/2021/4395228)
Supplement: Supplementary Materials — Supplementary Description: 1. Supplemental File 1 search strategy in PubMed. 2. Supplemental File 2 forest plot of HDL-C. 3. Supplemental File 3 forest plot of CRP. [file 4395228.f1.zip › 4395228.f1/Supplementary Description.docx]

Supplementary Description:

1. Supplemental File 1 Search strategy in PubMed
2. Supplemental File 2 Forest plot of HDL-C
3. Supplemental File 3 Forest plot of CRP
